# MicroRNA-320a Promotes Epithelial Ovarian Cancer Cell Proliferation and Invasion by Targeting RASSF8

**DOI:** 10.3389/fonc.2021.581932

**Published:** 2021-02-25

**Authors:** Lili Zhang, Huixiao Chen, Fengxi He, Shiqian Zhang, Aihua Li, Aifeng Zhang, Anqi Zhang

**Affiliations:** ^1^ Department of Obstetrics and Gynecology, Liaocheng People’s Hospital, Liaocheng, China; ^2^ Shandong University, Jinan, China; ^3^ Department of Obstetrics and Gynecology, Qilu Hospital of Shandong University, Jinan, China; ^4^ Department of Obstetrics and Gynecology, Tongzhou Maternal and Child Health Hospital of Beijing, Beijing, China

**Keywords:** microRNA-320a, proliferation, invasion, RASSF8, EOC

## Abstract

MicroRNAs (miRNAs) play important roles in tumorigenesis by controlling target gene expression. With opposing roles as a tumor suppressor or oncogene, microRNA-320a (miR-320a) was found to participate in tumor genesis and progression and also identified as a potentially useful marker in cancer diagnosis, treatment, and prognosis. To better understand the role of miR-320a in ovarian cancer, we investigated miR-320a expression in epithelial ovarian cancer (EOC) specimens as well as EOC cell lines and analyzed correlations between miR-320a expression and processes associated with EOC progression. The miR-320a level in EOC specimens was found to be associated with ovarian cancer progression and infiltration. Through *in vitro* and *in vivo* studies, we found that miR-320a significantly promoted the proliferation, migration, and invasion of EOC cells, and we identified RASSF8 as a target gene of miR-320a that was downregulated in EOC tissues and cell lines. *In vitro* downregulation of RASSF8 promoted the growth, migration, and invasion of EOC cells. Together these findings indicate that RASSF8 is a direct target of miR-320a, through which miR-320a promotes the progression of EOC.

## Introduction

Epithelial ovarian cancer (EOC) is the most lethal subtype of this gynecologic malignancy (1), with a 5-year survival rate of approximately 35–40% (2). The poor prognosis has generally been attributed to late diagnosis and chemoresistance-associated relapse (1, 3).

The functions of microRNAs (miRNAs) in cancer have been intensively explored in recent years, and various miRNAs have been shown to be of great importance in tumor initiation, progression, and metastasis. Furthermore, some miRNAs represent potential therapeutic targets for cancer management. miRNAs are a class of small, non-coding RNAs (18–22 nt in length) that bind to the 3’-untranslated regions (3’-UTRs) of the mRNAs of target genes, resulting in mRNA degradation and thereby regulating target gene expression (4). Prior research has shown altered expression of miR-320a in various cancers, such as salivary adenoid cystic carcinoma (5), colon cancer (6), gastric cancer (7) and breast cancer (8). However, the potential role of miR-320a in EOC remained to be determined. By analyzing ovarian cancer-related data from the Gene Expression Omnibus (GEO), Wang et al (9). identified 17 significantly differentially expressed miRNAs in ovarian cancer, and among them, miR-320 expression was found to correlate with poor prognosis.

The present study aimed to elucidate the mechanism by which miR-320a influences EOC progression. We analyzed miR-320a expression in clinical EOC specimens as well as EOC cell lines and investigated correlations between miR-320a expression and processes related to EOC progression. We then searched for target genes of miR-320a through which the effects of this miRNA on EOC may be mediated.

## Material and Methods

### Cell Culture

We obtained three ovarian cancer cell lines and one normal ovarian cell line from American Type Culture Collection (ATCC): SKOV3, OVCAR3, A2780, and IOSE80. These cells were maintained in a humidified incubator with 5% CO_2_ at 37°C in medium supplemented with 10% fetal bovine serum (FBS) (Invitrogen, Carlsbad, CA, USA). The cell culture media were McCoy’s 5A (Gibco, Carlsbad, CA, USA) for SKOV3 cells, RPMI-1640 (Gibco) for OVCAR3 and A2780 cells, and Dulbecco’s Modified Eagle’s Medium (DMEM, Hyclone, Logan, UT, USA) for IOSE80 cells.

### Clinical Specimens

Ovarian carcinoma tissue specimens and adjacent normal ovarian tissues were obtained from 40 patients who underwent initial hysterectomy at Liaocheng People’s Hospital between May 2014 and October 2016. All specimens were stored at –80°C.

Separately, ovarian tissue specimens were obtained from 60 patients diagnosed with EOC (aged 35–70 years with median age of 50 years). The EOC patients underwent cytoreductive surgery at Liaocheng People’s Hospital between January 2009 and December 2012. We collected the clinicopathological information of the patients including age, histological type, International Federation of Gynecology and Obstetrics (FIGO) 2014 staging, pathological grade, and presence of lymph node metastasis.

The present study was approved by the ethics committee of Liaocheng People’s Hospital (Liaocheng, China). Written informed consent was obtained from all participants prior to surgical treatment.

### RNA and miRNA Extraction and RT-PCR

Total RNA was extracted from cell lines and tissue samples using Trizol (Sangon Biotech, Shanghai). Reverse transcription was conducted by PrimeScript RT Master Mix Perfect Real Time (TaKaRa, Shiga, Japan) for miR-320a (forward: 5’-CGACGGAAAAGCTGGGTTGAGA-3’; reverse: 5’-ATCCAGTGCAGGGTCCGA-3’GG), miR-320a RT (5’-GTCGTATCCAGTGCAGGGTCCGAGGTATTCGCACTGGATACGACTCGCCC-3’), RASSF8 (forward: 5’-CATTCAAGGCCAGCAGAGTC-3’; reverse: 5’-TGCCGCAACTCCTTAGTCAA-3’). The expression level of miR-320a was normalized by that of U6 small nuclear RNA (RNU6B, Applied Biosystems, Foster City, CA, USA) by the 2^-ΔCT^ method; RASSF8 expression was normalized to that of the internal loading control glyceraldehyde-3-phosphate dehydrogenase (GAPDH, forward: 5’-GGAGCGAGATCCCTCCAAAAT-3’; reverse: 5’-GGCTGTTGTCATACTTCTCATGG-3’). PCR was applied using the RNA PCR Kit (TaKaRa) on a real-time PCR system (Applied Biosystems 7500).

### Transient Transfection

The miR-320a mimics and inhibitor as well as the corresponding negative controls along with siRNA against human RASSF8 (RNAi-a, 5’-CACCAAACGCTTACAGGACAA -3’; RNAi-b, 5’-GAAGAGG AAATTGTCCGTCTA-3’) and its negative control were all obtained from GenePharma (Shanghai, China). Transfection was conducted with Lipofiter (HanBio, Shanghai, China) when cells reached 70–80% confluency.

### Stable Transfection With Lentivirus

Plasmids pLenO-DCE-GFP+Puro with hsa-miR-320a mimics or control oligonucleotides, LV-miR-320a, or LV-miR-NC (negative control) were constructed by GenePharma. Lentivirus transfection was performed to establish stable EOC cell clones expressing miR-320a (LV-SKOV3/miR320a) and the control clones (LV-SKOV3/miR-NC). miR-320a expression was examined by qRT-PCR and normalized by U6 RNA expression. Similarly, RASSF8 expression was examined and normalized by GAPDH expression.

### Protein Extraction and Immunoblotting

Total protein was extracted using radioimmunoprecipitation assay (RIPA) lysis buffer (Vazyme Biotech, Nanjing, China) with phenylmethylsulfonyl fluoride (PMSF, Roche Applied Science, Basel, Switzerland). Western blotting was performed with primary antibodies against RASSF8 (1:1,000, Santa Cruz Biotechnology, Santa Cruz, CA, USA), vimentin (1:1,000, Cell Signaling Technology, Danvers, MA, USA), E-cadherin (1:1,000, Cell Signaling Technology), and β-actin (1:1,000, Abmart, Shanghai, China) as a loading control. The secondary antibody was goat anti-rabbit IgG conjugated with horseradish peroxidase (1:3,000, WanleiBio, Shenyang, China). Protein bands were separated and imaged using an enhanced chemiluminescence system (ProteinSimple, San Jose, CA, USA). Quantitative evaluation was conducted on Quantity One software version 4.0.1 (Bio-Rad Laboratories, Inc., Hercules, CA, USA). All experiments were conducted in triplicate.

### Plasmid Construction and Luciferase Assay

miR-320a-binding sites were predicted using TargetScan, mirbase, and miRTarBase. The pmirGLO vector contained the target gene as well as firefly and Renilla luciferase reporter genes (ThermoFisher, Waltham, MA, USA). The target gene, wild-type or mutant RASSF8 mRNA, was integrated into this dual-luciferase report gene system at the restriction sites MluI and HindIII. Cells were cotransfected with miRNA mimics (GenePharma, 50 nmol/L) and reporter vectors (0.2 μg/mL) using Lipofectamine 2000 (Invitrogen). After 24 h of transfection, luciferase activity was measured according to the manufacturer’s instructions with the Dual-Glo™ Luciferase Assay kit (Promega, Madison, WI, USA). Data are presented as mean ± standard deviation (SD) from one representative of triplicate experiments.

### Cell Proliferation Assay

The water-soluble tetrazolium salt (WST) assay and Cell Counting Kit-8 (Dojindo, Kumamoto, Japan) were used to evaluate cell proliferation according to the manufacturer’s instructions. Cell viability was quantified by colorimetric analysis on a microplate reader (Thermo Fisher Scientific).

### Transwell Invasion Assay

Cell invasion and migration activity were assessed by a Transwell invasion assay. Briefly, 3×10^5^ cells were seeded in 100 µl serum-free medium and incubated in an upper chamber with or without basement membrane matrix (BD Bioscience, Bedford, MA, US), and medium with 20% FBS was placed in lower chambers. After 24 h, the cells were gently removed from the upper chamber with a cotton swab, and cells on the bottom of the insert were stained with 1% crystal violet for 30 min, before counting of the numbers of cells in five random fields. The relative invasion and migration results are presented as mean ± SD.

### Apoptosis Assay

Cell apoptosis was examined using an Annexin V-APC/7-AAD Apoptosis Detection Kit (BD Biosciences, San Jose, CA, USA) and analyzed by flow cytometry using the BD FACSAria instrument.

### *In Vivo* Tumor Growth Assay

The *in vivo* experiment was approved by the Institutional Animal Care and Use Committee of the Shandong University. We injected LV-SKOV3/miR-320a cells or LV- SKOV3/miR-NC cells (3.3×10^7^/ml) subcutaneously into male nude mice (5 weeks old, n=5 per group) at their left flank. Tumor growth was represented by volume (mm^3^, V=tumor length×width^2^/2) measured every 2–3 days (10). At 5 weeks after inoculation, mice were euthanized, and the tumors were removed.

### Immunohistochemistry (IHC)

Excised tumor tissues were fixed, embedded, and stained with hematoxylin and eosin (HE) (Sigma-Aldrich, St. Louis, MO, USA). Immunostaining with anti-vimentin (1:200, Cell Signaling Technology) and anti-E-cadherin (1:200, Cell Signaling Technology) was also performed, according to methods described previously (10). Sections were stained with 2-Solution DAB (ZSJQ-Bio, Beijing, China) and Mayer’s hematoxylin (Sigma-Aldrich). The staining intensity was classified as negative (none-to-weak staining) or positive (moderate-to-strong staining). Sections processed similarly except without the primary antibodies serve as negative controls.

### Statistical Analysis

The statistical significance of differences in the data was determined by Student t test and one-way analysis of variance (ANOVA) in SPSS 16.0 (SPSS Inc., Chicago, IL, USA). A two-tailed p value <0.05 indicated significant difference.

## Results

### miR-320a *I*s Upregulated in Ovarian Cancer Samples and Cell Lines

**Figure 1:**
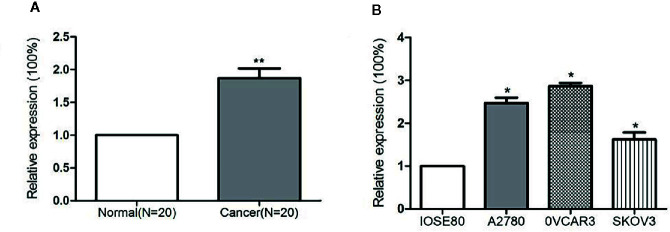
miR-320a expression is upregulated in EOC samples and cell lines. **(A)** Expression of miR-320a mRNA among 20 paired ovarian tissues (RT-PCR), showing higher miR-320a mRNA expression in EOC tumors than in adjacent normal ovarian tissues. **(B)** miR-320a mRNA expression in three ovarian cell lines (RT-PCR) was greater than that in IOSE80 normal ovarian cells. (**p < 0.01; *p < 0.05).

miR-320a expression levels in 20 pairs of EOC tissues and adjacent normal ovarian tissues, in three ovarian cancer cell lines, and in a normal ovarian epithelial cell line were determined by qRT-PCR. The level of miR-320a expression in EOC tissues was significantly higher than that in normal tissues (**Figure 1A**). Moreover, miR-320a expression was higher in the three ovarian cancer cell lines than in the normal ovarian epithelial cell line (**Figure 1B**).

To investigate how miR-320a expression may correlate with clinical features and clinicopathologic factors in EOC, we measured miR-320a expression in 60 primary EOC tissues by qRT-PCR. The miRNA-320a levels were categorized as low or high, using the median miRNA-320a level as the cut-off value. We found that miR-320a positivity was correlated with the FIGO stage of patients as well as lymph node metastasis, but not with histological type, pathologic grade, or age (**Table 1**). Based on these data, we presume that miR-320a acts as an oncogene in EOC progression.

**Table 1:** Correlations between miR-320a expression and the clinicopathological features of ovarian cancer.

Clinical pathology	n	miR-320a expression, n (%)			χ^2^	p
		Low (30)	High (30)			
Age (years)						
≤50	14	9	5			
>50	46	21	25		1.491	0.180
Histologic type						
Serous	35	17	18			
Endometrioid	25	13	12		0.069	0.500
FIGO Stage						
I+II	18	13	5			
III+IV	42	17	25		5.079	0.024
Pathologic grade						
G1	11	6	5			
G2+G3	49	24	25		0.111	0.500
Lymph node metastasis						
Yes	45	17	28		10.756	0.001
No	15	13	2			

FIGO, International Federation of Gynecology and Obstetrics. The 2014 FIGO staging was used.

### miR-320a Promotes EOC Cell Proliferation and Migration *In Vitro*

**Figure 2:**
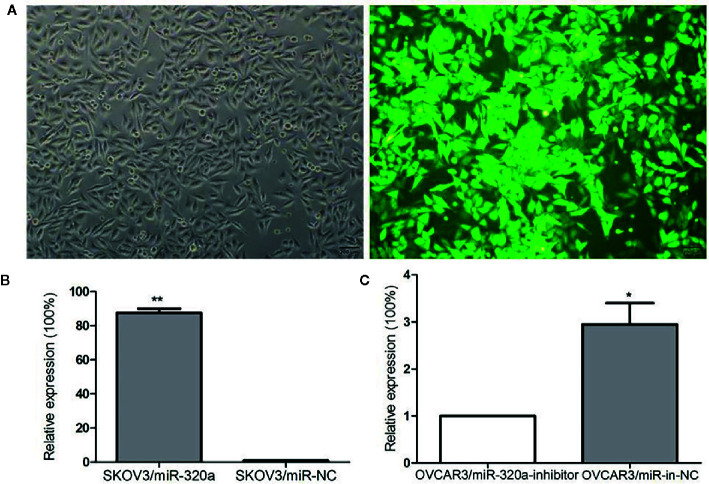
miR-320a transfection efficiency. **(A)** Stable transfection by lentivirus and the fluorescence infection efficiency in SKOV3 cells was almost 100% under fluorescence microscopy. Scale bars, 200 µm. **(B, C)** Transfection efficiencies of miR-320a in SKOV3 and OVCAR3 cells by qRT-PCR. (*p < 0.05; **p < 0.01).

To explore the effects of miR-320a expression on EOC cell growth, we transfected the SKOV3 cells, which expressed the lowest level of miR-320a among the EOC cell lines, with LV-miR-320a mimics to establish stable miR-320a-overexpressing clones (SKOV3/miR-320a); SKOV3 cells transfected with LV-miR-NC were used as the control. We also transiently transfected OVCAR3 cells, which expressed the highest level of miR-320a among the EOC cell lines, with miR-320a inhibitor (OVCAR3/miR-320a-inhibitor); OVCAR3 cells expressing miR-in-NC served as the control (**Figure 1B**). MicroRNA mimics with fluorescent labels were used to determine the transfection efficiency (**Figure 2A**), which reached almost 100%. Moreover, qRT-PCR showed that miR-320a expression in SKOV3/miR-320a cells was significantly higher than that in SKOV3/miR-NC cells (**Figure 2B**), and OVCAR3/miR-320a-inhibitor cells expressed a lower level of miR-320a than OVCAR3/miR-in-NC cells (**Figure 2C**).

**Figure 3:**
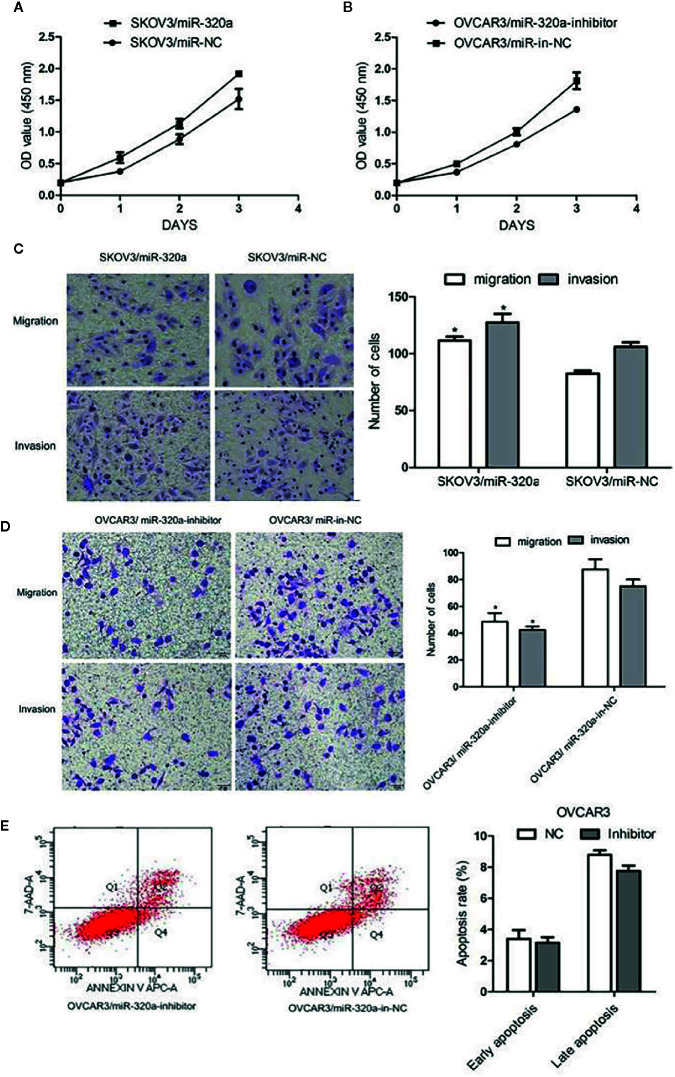
miR-320a promotes EOC cell proliferation, migration, and invasion *in vitro*. **(A, B)** miR-320a expression promoted EOC cell proliferation. **(C, D)** miR-320a promoted EOC cell migration/invasion. **(E)** miR-320a expression has no effect on apoptosis of EOC cells. (*p < 0.05).

In SKOV3 cells, miR-320a overexpression promoted cell growth and increased migration and invasion (**Figures 3A, C** **)**. In OVCAR3 cells, miR-320a downregulation slowed proliferation and impaired cell migration and invasion (**Figures 3B, D** **)**. Compared to the respective control, miR-320a downregulation in OVCAR3 cells had no obvious effect on apoptosis (**Figure 3E**). Taken together, these results suggest that miR-320a expression promotes EOC cell proliferation and migration/invasion *in vitro*.

### miR-320a Promotes Tumor Growth *In Vivo*

**Figure 4:**
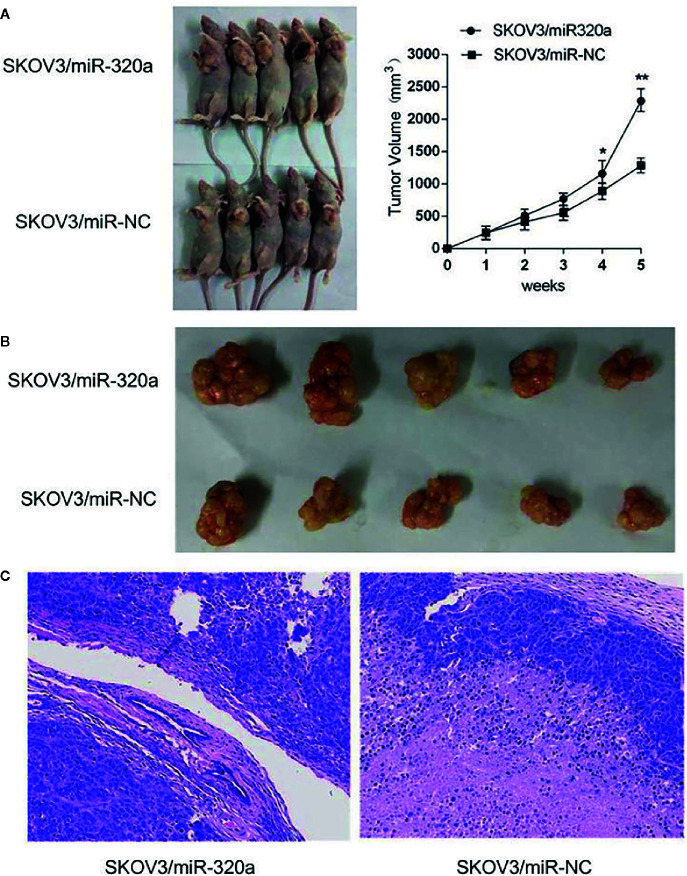
miR-320a promotes tumorigenesis of SKOV3 cells *in vivo*. **(A, B)** The tumor volume in the SKOV3/miR-320a group was significantly greater than that in the control group. **(C)** Tumor sections were stained with H&E, and tumor cell invasion was observed in the tumors produced from the SKOV3/miR-320a cells. Magnification: 10×. (*p < 0.05, **p < 0.01).

To explore the oncogenic role of miR-320a, an *in vivo* study was conducted in a mouse model, with tumor volume and miR-320a expression (indicated by green fluorescent protein [GFP] expression) as the main measurements. Compared to mice injected with SKOV3/miR-NC control cells, those inoculated with miR-320a–overexpressing SKOV3 cells showed increased tumor growth (**Figure 4A**) and tumor size (**Figure 4B**). In addition, tumor sections stained by H&E showed tumor cell invasion in the SKOV3/miR-320a cell-derived tumors (**Figure 4C**). These findings indicate that miR-320a promotes EOC cell growth *in vivo*.

### miR-320a Promotes Epithelial–Mesenchymal Transition in Ovarian Cells

**Figure 5:**
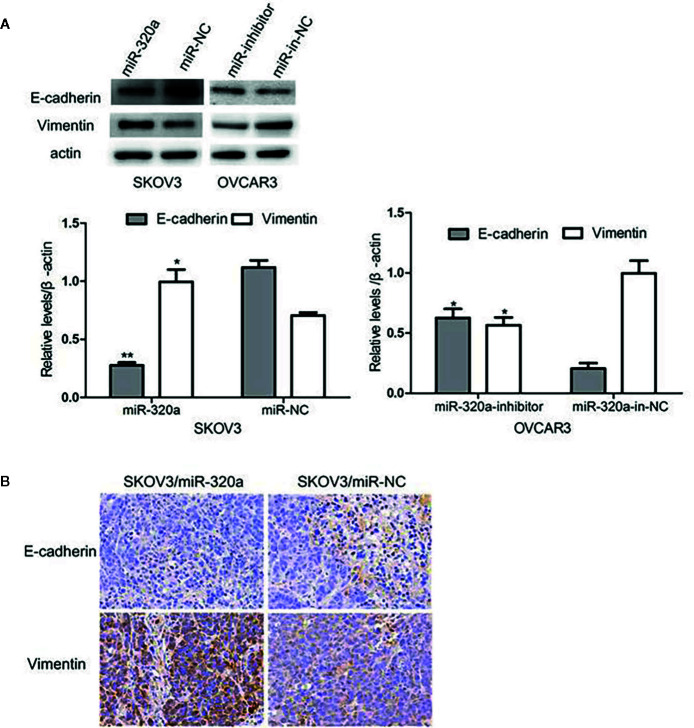
miR-320a can regulate EMT in EOC cells *in vitro* and *in vivo*. **(A)** miR-320a downregulated the expression of E-cadherin and upregulated the expression of vimentin in EOC cells. **(B)** EMT marker expression in nude mice tumors. Immunohistochemistry analysis showed that E-cadherin expression was increased while vimentin was reduced in the SKOV3/miR-NC tumors compared to the SKOV3/miR-320a tumors. (*p < 0.05, **p < 0.01).

EMT is a crucial process in oncogenesis (11–13). Given that miR-320a promoted SKOV3 cell migration and tumor formation, we presumed that miR-320a may participate in EMT. In SKOV3/miR-320a cells, expression of the epithelial cell marker E-cadherin was significantly reduced, while the expression of the mesenchymal cell marker vimentin was increased (**Figure 5A**), which indicated enhanced EMT in EOC cells overexpressing miR-320a. Moreover, compared to tumor tissue formed from control cells, the tumor tissue formed from SKOV3/miR-320a cells in mice showed less E-cadherin staining and higher vimentin signal (**Figure 5B**). Therefore, both *in vitro* and *in vivo* studies confirm an EMT-promoting effect of miR-320a in EOC.

### RASSF8 Is a Direct Target of miR-320a

**Figure 6:**
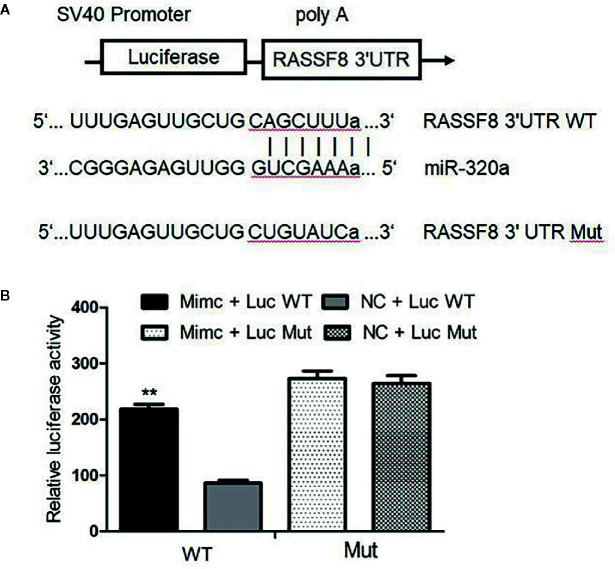
Luciferase assay confirmed that RASSF8 is a target gene of miR-320a. **(A)** Schematic diagram of the binding of miR-320a to the target site in the RASSF8 3 ‘UTR. **(B)** A double luciferase reporter gene was used to detect the interaction between miR-320a and the RASSF8 3’UTR. (**p < 0.01).

miRNAs usually bind and downregulate their target genes to exert functions. To identify the possible functional targets of miR-320a and to investigate the mechanisms of miR-320a-induced migration and invasion suppression, we searched for targets of miR-320a in TargetScan, mirbase and miRTarBase and finally identified four genes: RASSF8, DNAJA2, GOLT1B, and PTEN. The present study focused on the RASSF8 gene. The double luciferase reporter gene vector pmirGLO was used to construct the recombinant plasmids pGL-RASSF8 3’UTR-WT for the wild-type RASSF8 gene and pGL-RASSF8 3’UTR-Mut for a mutant RASSF8 gene with mutation in the 3’UTR region. In SKOV3 cells, the fluorescence signal after miR-320a mimics+pGL-RASSF8 3’UTR-WT co-transfection was higher than that after NC+RASSF8 3’UTR-WT co-transfection, whereas no significant difference in fluorescence signal was found between cells transfected with the pGL-RASSF8 3’UTR-Mut and NC+RASSF8 3’UTR-Mut (**Figures 6A, B** **)**. These data suggest that miR-320a may regulate RASSF8 gene expression upon binding to its 3’UTR region.

### miR-320a Downregulates RASSF8 Expression

**Figure 7:**
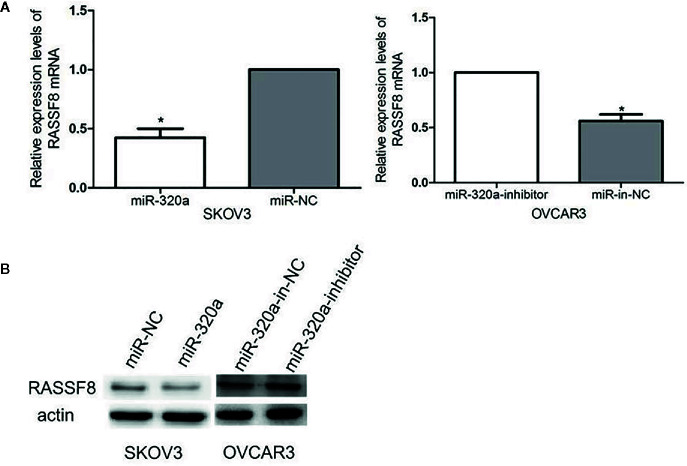
**(A, B)** miR-320a expression reduced the mRNA and protein levels of RASSF8. (*p < 0.05).

We evaluated RASSF8 mRNA and RASSF8 protein levels in EOC cells lines by qRT-PCR and western blotting, respectively. We found that miR-320a-overexpressing SKOV3 cells exhibited significantly downregulated RASSF8 expression, compared with the negative control group (**Figures 7A, B** **)**. Similarly, OVCAR3 cells in which miR-320a was downregulated showed higher RASSF8 mRNA and protein levels than control OVCAR3 cells (**Figures 7A, B** **)**. These results suggest that miR-320a can negatively regulate RASSF8 expression in EOC, which also confirms that RASSF8 is a target gene of miR-320a.

### RASSF8 Downregulation Promotes EOC Cell Growth and Migration *In Vitro*

**Figure 8:**
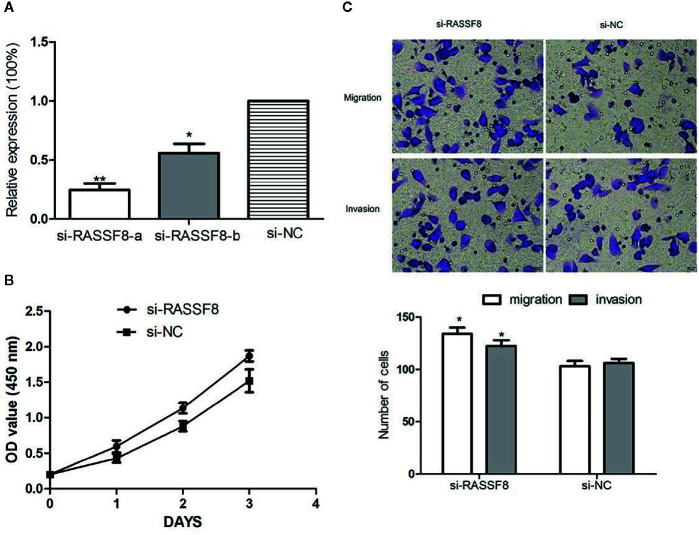
Downregulation of RASSF8 promoted the proliferation, migration, and invasion of EOC cells. **(A)** The inhibition efficiency in the si-RASSF8-a group was significantly lower than that in the normal control group. **(B)** Downregulation of RASSF8 promoted the proliferation of EOC cells. **(C)** Downregulation of RASSF8 promoted the migration/invasion of EOC cells. (*p < 0.05, **p < 0.01).

To explore the role of RASSF8 in EOC cells, SKOV3 cells were transfected with one of two RASSF8 siRNAs (RASSF8-RNAi-a or RASSF8-RNAi-b) or the negative control (si-NC). SKOV3 cells transfected with either RASSF8-RNAi-a or RASSF8-RNAi- showed reduced RASSF8 mRNA expression, but the greatest reduction was seen with RASSF8-RNAi-a (**Figure 8A**). Next we observed that RASSF8-RNAi-a–transfected cells (si-RASSF8) exhibited enhanced proliferation and migration compared with control cells (**Figures 8B, C** **)**. These observations suggest that RASSF8 inhibits the proliferation, invasion, and migration in EOC cells *in vitro*.

## Discussion

Regulatory roles of miRNAs in human cancer have been gradually revealed along with the potential of miRNAs as therapeutic targets. EOC is a major cause of tumor-related death in females, and in research related to EOC progression and metastasis, some miRNAs have been shown to be oncogenic while others were shown to have suppressive functions (9, 14–16). The present study identified miR-320a as upregulated in EOC with a potential target gene being RASSF8. The results indicate that miR-320a may accelerate tumor progression by downregulating RASSF8.

miR-320a expression has been identified in a diverse variety of human tumors (5, 6, 8, 17–19). miR-320a acts as a tumor suppressor in some cancers and as an oncogene in other types, potentially according to cellular background. The present study was conducted to determine the role of miR-320a in EOC. We observed that EOC tissues and cell lines exhibited higher miR-320a expression than normal tissues and cell lines. When we induced miR-320a overexpression in EOC cells, the cells showed increased proliferation and migration. Accordingly, we hypothesize that miR-320a promotes ovarian cancer progression, which is consistent with the conclusion of Wang et al. (9). Moreover, we found that miR-320a may regulate EMT. In SKOV3 cells, miR-320a overexpression upregulated the mesenchymal marker vimentin and downregulated the epithelial marker E-cadherin. Additionally, miR-320a downregulation in OVCAR3 cells promoted E-cadherin expression and reduced vimentin expression. Promotion of EMT by miR-320a indicates a carcinogenic function of miR-320a in EOC, and the underlying mechanism is of great interest and worth further investigation.

Our bioinformatics analysis identified RASSF8 as a potential downstream target gene of miR-320a and that miR-320a regulates RASSF8 expression by directly binding to the 3’-UTR of RASSF8 mRNA. This predication was confirmed by increased luciferase activity and RASSF8 protein expression induced by miR-320a. The members of the RAS-association domain family (RASSF) are involved in various key activities, such as cell proliferation, microtubule stability, apoptosis, promoter methylation, and vesicle trafficking, and they have been recognized as potential tumor suppressors (20, 21). RASSF8 has been described as a potential tumor suppressor in lung carcinogenesis and cervical cancer (9, 22, 23). Lock et al. (22) reported that RASSF8 is important in maintaining adherens junction activity and cell migration in epithelial cells. To investigate the function of RASSF8, we used RNA interference to knockdown RASSF8 gene expression in EOC cells and found that RASSF8 inhibited the proliferation and migration of EOC *in vitro*. Moreover, RASSF8 protein expression was greatly suppressed by overexpression of miR-320a, which is consistent with previous reports (9, 22, 23). Overall, our results indicate that miR-320a may promote EOC progression upon acting on RASSF8 and disrupting its function.

In summary, the results of the present study revealed that miR-320a expression is upregulated in EOC tissues and cell lines. This study provides novel evidence that miR-320a negatively targets the tumor suppressor gene RASSF8 and promotes the proliferation of EOC cells both *in vitro* and *in vivo*. Therefore, miR-320a and its target RASSF8 are attractive candidates for future studies to develop novel methods for EOC diagnosis and treatment.

## Data Availability Statement

The original contributions presented in the study are included in the article/supplementary materials. Further inquiries can be directed to the corresponding authors.

## Ethics Statement

The studies involving human participants were reviewed and approved by the ethics committee of Liaocheng People’s Hospital (Liaocheng, China). The patients/participants provided their written informed consent to participate in this study.

## Author Contributions

LZ and HC contributed to the conception of the work and designing the study. AFZ and AQZ searched the literature and collated the data. LZ and FH performed the experiments. LZ analyzed the data and drafted the manuscript. SZ and AL made substantial contributions to the analysis and interpretation of data, and revised the manuscript critically for important intellectual content. All authors contributed to the article and approved the submitted version.

## Funding

The present study was supported by the Medical and Health Technology Development Program in Shandong Province (grant No. 2017WS641). The funder had no role in the design of the study, in the collection, analyses, or interpretation of data, in the writing of the manuscript, or in the decision to publish the results.

## Conflict of Interest

The authors declare that the research was conducted in the absence of any commercial or financial relationships that could be construed as a potential conflict of interest.
